# Can Proprioceptive Training Reduce Muscle Fatigue in Patients With Motor Neuron Diseases? A New Direction of Treatment

**DOI:** 10.3389/fphys.2019.01243

**Published:** 2019-10-01

**Authors:** Ayman A. Mohamed

**Affiliations:** Department of Physiotherapy and Rehabilitation, School of Health Sciences, Istanbul Gelisim University, Istanbul, Turkey

**Keywords:** motor neuron diseases, muscle fatigue, older patients, proprioceptive, training

## Abstract

Muscle fatigue is a serious problem in patients with motor neuron diseases (MNDs). It commonly disturbs both daily life activity and rehabilitation tolerance. A particular concern should be taken when MNDs occur in older ages. Older patients with MNDs usually have a worse clinical presentation and a lower survival rate. This could increase the occurrence of muscle fatigue. Muscle fatigue occurs due to a dysfunction in either motor or sensory systems. Current exercise interventions performed to decrease the occurrence of muscle fatigue focused only on treating motor causes of muscle fatigue. It has been demonstrated that these interventions have a high debate in their effectiveness on decreasing the occurrence of muscle fatigue. Also, these exercise interventions ignored training the affected sensory part of muscle fatigue, however, the important role of the sensory system in driving the motor system. Thus, this review aimed to develop a novel exercise intervention by using proprioceptive training as an intervention to decrease the occurrence of muscle fatigue in patients with MNDs particularly, older ones. The physiological effects of proprioceptive training to decrease the occurrence of muscle fatigue could include two effects. The first effect includes the ability of the proprioceptive training to increase the sensitivity of muscle spindles as an attempt to normalize the firing rate of α-motoneurons, which their abnormalities have major roles in the occurrence of muscle fatigue. The second effect includes its ability to correct the abnormal movement-compensations, which develop due to the biomechanical constraints imposed on patients with MNDs.

## Introduction

Muscle fatigue is a common problem in patients with motor neuron diseases (MNDs) (Gibbons et al., 2013). MNDs are defined as a group of diseases in which there is a progressive degeneration of motor neurons (Quansah and Karikari, 2015). MNDs include three main subtypes. The first subtype includes disorders which affect lower motor neurons, such as spinal muscular atrophy (SMA) and spinobulbar muscular atrophy (SBMA or Kennedy’s disease). The second subtype includes disorders which affect upper motor neurons, such as spastic paraplegias and primary lateral sclerosis (PLS). The last subtype includes disorders which affect both upper and lower motor neurons, such as amyotrophic lateral sclerosis (ALS) (Figlewicz and Orrell, 2003).

One of the common complaints in patients with MNDs is muscle fatigue (McElhiney et al., 2009; Abraham and Drory, 2012; Gibbons et al., 2018). Muscle fatigue is defined as the time-related reduction in the maximum force-production capacity of the muscle. Muscle fatigue is one of the most common complaints occur in patients with MNDs. McElhiney et al. (2009) have demonstrated that muscle fatigue affects approximately 44% of patients with MNDs. It has been reported that there are several causes for the occurrence of muscle fatigue in MNDs. Most studies have demonstrated that neurodegenerative cause is the main cause of muscle fatigue in patients with MNDs, while the neuromuscular transmission, and muscle metabolism are normal in those patients (Abraham and Drory, 2012). In contrast, other studies have stated that patients with ALS usually have a neuromuscular junction disassembly and muscle denervation and this abnormality of the neuromuscular junction is trademark feature of ALS onset and progression. Other studies have shown that there is a severe abnormality in the mitochondrial function in patients with ALS (Bowling et al., 1993; Dupuis et al., 2005; Vandoorne et al., 2018).

However, MNDs are common in adults, this previous debate increases in older ages because older patients with MNDs usually experience more complex clinical presentation and lower survival rate (Eisen et al., 1993; Forbes et al., 2004; Broussalis et al., 2018). It has been demonstrated that the prognosis is much worse in older patients with MNDs than younger ones (Tysnes et al., 1994). Older patients with MNDs usually have a higher incidence of bulbar symptoms than younger ones and this bulbar onset presents in at least half of all patients with MNDs over 80 years (Eisen et al., 1993; Forbes et al., 2004; Broussalis et al., 2018). Also, older patients with MNDs could experience more occurrence of muscle fatigue during exercises than younger ones because aging produces an abnormality in normal function and firing rate of the α-motoneurons (Soderberg et al., 1991; Knight and Kamen, 2007; Watanabe et al., 2016), which is considered one of the main causes of muscle fatigue.

Furthermore, it has been shown that elderlies usually experience a high degree of muscle fatigue during exercise rather than younger ones (Klass et al., 2007; Mcneil and Rice, 2007; Kent-Braun, 2009). Klass et al. (2007) investigated the effect of aging on the fatigability of ankle dorsiflexor muscles throughout concentric and eccentric contractions. They found that fatigability increased progressively with aging and they argued this increase in fatigability to peripheral alterations occurred in Ca^2+^-controlled excitation-contraction coupling process and neuromuscular propagation. Thus, the occurrence of MNDs in older ages could cause further alteration in the Ca^2+^-controlled excitation-contraction coupling process and neuromuscular propagation.

Moreover, it has been demonstrated in the literature that there is a change in muscle fibers with both aging and MNDs. With aging, type I muscle fibers usually transform to type II, which increases the occurrence of muscle fatigue during exercises (Mcneil and Rice, 2007; Kent-Braun, 2009). Also, it has been shown that with MNDs, there are atrophic changes in muscle fibers with mild denervation. Thus, older patients with MNDs could experience more changes in muscle fibers than younger ones due to the combination of aging and MNDs effects. Thus, older patients with MNDs should not be neglected from future revisions.

Current exercise interventions performed to treat muscle fatigue in patients with MNDs are few and their qualities are very low (Gibbons et al., 2018). Thus, it is impossible to reach strong conclusions about the effectiveness of these interventions to reduce the occurrence of muscle fatigue in patients with ALS/MND (Gibbons et al., 2018). Generally, exercise interventions performed to treat muscle fatigue have assumed that muscle fatigue occurs due to dysfunction in motor control. This dysfunction occurs due to failure in one or more mechanisms included in the voluntary muscle contraction. This failure can occur in any area along the neuromuscular system, including the motor cortex, signals from the motor cortex to motoneurons, signals from motoneurons to muscle, neuromuscular junction coupling in muscle, or actin-myosin links (Light et al., 2010).

Also, current exercise interventions included either grading exercise intensity (Wallman et al., 2004), increasing rest period (Nogueira et al., 2012), using mild training intensity (Dennett et al., 2016), or using massage for the fatigued muscle (Nunes et al., 2016). The effectiveness of these exercise interventions is still in debate. Some studies (Morriss et al., 1996; Friedberg, 2002; Wallman et al., 2004) have demonstrated that increasing physical activity or grading exercise intensity is beneficial in decreasing the occurrence of muscle fatigue. In contrast, other studies (Black et al., 2005; Oh et al., 2016) have shown that increasing physical activity or grading exercise intensity has no effect on reducing the occurrence of muscle fatigue.

It has been shown in the literature that muscle fatigue does not occur due to dysfunction in the motor control only, however, it occurs due to dysfunction in both motor and sensory systems (Light et al., 2010). Several studies have shown that MNDs affect sensory neurons besides motor neurons (Anagnostou et al., 2005; Pugdahl et al., 2007; Vaughan et al., 2015). Pugdahl et al. (2007) conducted a study to detect the presence of any dysfunction in sensory neurons in patients with ALS. They found that about 22.7% of the included patients had an abnormality in the conduction time of at least one sensory nerve. Anagnostou et al. (2005) conducted a study to detect the presence of any dysfunction in sensory neurons in children with SMA. They found that children with SMA had dysfunctions in the conduction time of sensory nerves. Recently, Vaughan et al. (2015) conducted a study to detect the presence of any dysfunction of the proprioceptive system in mice with ALS. They found that these mice had significant degenerations in nerve endings of the proprioceptive system.

Also, it has been demonstrated in several studies that muscle fatigue has sensory receptors responsible for sensing and developing muscle fatigue (Light et al., 2010; Boyas and Guével, 2011; Staud, 2012; Nunes et al., 2016; Kuppuswamy, 2017). However, the vital role of the sensory system in driving motor control (Riemann and Lephart, 2002), till now there is no exercise intervention focused on improving the function of the sensory element of muscle fatigue. It has been shown in the literature that the sensory mechanism of muscle fatigue starts from mechanoreceptors and metaboreceptors. These receptors are responsible for generating the sensation of muscle fatigue (St Clair Gibson et al., 2003; Light et al., 2010). Mechanoreceptors are primary receptors of muscle fatigue and they are sensitive to changes in muscle strain (St Clair Gibson et al., 2003; Light et al., 2010). While metaboreceptors are secondary receptors of muscle fatigue and they are sensitive to changes in the number of metabolites created by muscle contraction (St Clair Gibson et al., 2003; Light et al., 2010).

Mechanoreceptors are also the same receptors of the proprioception. The sensitivity and function of mechanoreceptors can be improved both neurologically or morphologically by performing proprioceptive training (Kaya, 2016). Thus, it is reasonable to suppose that performing a proprioceptive training to the fatigued muscle could improve the function of mechanoreceptors within this muscle. Consequently, this could be an effective modality to reduce the occurrence of muscle fatigue in patients with MNDs, particularly older patients who have a worse clinical presentation, and a low survival rate. Thus, this review aimed to demonstrate possible physiological mechanisms of proprioceptive training as an exercise intervention to treat muscle fatigue in patients with MNDs, particularly older ones.

This review included seven subtopics, the previous mechanisms of muscle fatigue in MNDs, the proprioception dysfunctions in MNDs, the mechanism of the sensation of muscle fatigue, the possible physiological effects of proprioceptive training on decreasing muscle fatigue in MNDs, the effects of proprioceptive training to correct the imposed biomechanical constraints in MNDs, the effects of aging on pathological degeneration of motor and sensory neurons in patients with MNDs, and the physiological effects of proprioceptive training to create theoretical bases to fight the MNDs in elderlies.

### The Previous Mechanisms of Muscle Fatigue in MNDs

It has been reported that muscle fatigue in MNDs occurs as a result of a defect in lower motor neurons. This defect causes a failure of motor units to provide the needed levels of activity, consequently, peripheral muscle fatigue occurs (Abraham and Drory, 2012). In the literature, neurodegenerative causes are the main causes of muscle fatigue. These neurodegenerative causes include any dysfunction of microglia, glutamate excitotoxicity, misfolded proteins, mitochondrial dysfunction, or oxidative stress (Abraham and Drory, 2012).

Vucic et al. (2007) investigated the alteration of axonal excitability occurred after an induced voluntary contraction to recognize peripheral mechanisms of muscle fatigue in patients with ALS. They found that patients with ALS had a membrane hyperpolarization. This membrane hyperpolarization caused an increase in the threshold occurred after the voluntary contraction in patients with ALS compared with controls. They argued this membrane hyperpolarization to the abnormality in either the Na^+^/K^+^ pump or firing rate of motor neurons. They also found that there was a dysfunction in the Na + /K + ATPase, which might cause a loss of motor neurons.

Sharma et al. (1995) examined possible mechanisms of muscle fatigue in patients with ALS. They measured muscle force, energy metabolism, and muscle activation pattern. They used the phosphorus-3 1 magnetic resonance spectroscopy to measure muscle force and energy metabolism, and the neurophysiological measures and magnetic resonance imaging to measure the muscle activation pattern. These measures were collected through a 25 min intermittent isometric contraction of the tibialis anterior muscle. They found that both tetanic and maximum voluntary force decayed in those patients more than controls. Also, they found that muscular activation impaired due to small proton signal intensities and amounts of energy metabolites. Lastly, they found that the neuromuscular transmission was nearly normal because amplitudes of the evoked compound of the muscle action potential were steady throughout the contraction.

However, the common belief that the neuromuscular transmission and muscle metabolism are normal in patients with MNDs, several studies have demonstrated that patients with MNDs experience abnormalities in the neuromuscular transmission, and mitochondrial function (Bowling et al., 1993; Dupuis et al., 2005; Rocha et al., 2013; Cappello and Francolini, 2017; Vandoorne et al., 2018). Cappello and Francolini (2017) have stated that patients with ALS usually have a neuromuscular junction disassembly and muscle denervation. Additionally, Rocha et al. (2013) have demonstrated that the degeneration of the neuromuscular junction is a trademark feature of ALS onset and progression. Also, It has been shown in several studies that there is a severe abnormality in the mitochondrial function in patients with ALS (Bowling et al., 1993; Dupuis et al., 2005; Vandoorne et al., 2018). Thus, this abnormality in the neuromuscular transmission and mitochondrial function should be considered as a source of muscle fatigue in patients with MNDs in the future.

### The Proprioception Dysfunctions in MNDs

Motor neuron diseases significantly disturb the whole proprioceptive system. Several studies have demonstrated that MNDs usually disturb a variety of cells, such as Renshaw and Glial cells in the spinal cord (Haidet-Phillips et al., 2011; Mochizuki et al., 2011; Philips and Rothstein, 2014; Vaughan et al., 2015). Some studies used neurophysiological and neuroimaging analyses to detect the presence of any abnormality in the sensory neurons in patients with ALS. They found that about 20–60% of sensory neurons showed an abnormality in those patients (Hammad et al., 2007; Pugdahl et al., 2007). Other studies used a histological analysis to detect any abnormality in the sensory neurons. Also, they found that there was a significant degree of degeneration in these neurons and their axons (Dyck et al., 1975; Hammad et al., 2007).

One of the major sensory systems which has a vital role in driving motor control is the proprioceptive system (Vaughan et al., 2015). The degeneration of proprioceptive neurons, mainly Ia/II proprioceptors, would possibly have a significant effect on increasing the deterioration of α-motoneurons. It has been demonstrated that proprioceptive (sensory) and α-motor neurons are structurally and functionally connected. Proprioceptive information detected by mechanoreceptors delivers to α-motoneurons via monosynaptic connections to adjust their actions. Thus, any loss of the proprioceptive mechanism could highly affect α-motoneurons function (Vaughan et al., 2015).

Two animal studies have demonstrated that MNDs significantly affect the proprioceptive mechanism. Mentis et al. (2011) conducted a study to detect the presence of any early signs of malfunction in the sensory-motor connectivity in mice with SMA. They found that mice with SMA experienced a decrease in proprioceptive reflexes and a decrease in function and number of proximal dendrites and motor neuron synapses. These abnormalities raised early through the disease and they accompanied the affection of motor neurons. Also, Vaughan et al. (2015) conducted a study to detect the presence of any degrees of degeneration in nerve endings of the proprioceptive system in mice with ALS. They found that peripheral nerve endings of the proprioceptive system experienced a significant degree of degeneration, particularly types Ia/II. This degeneration occurred early prior to the presence of any neurological symptoms or loss of any central projecting nerve branches.

To the best of our knowledge, there was no human study in the literature demonstrated the effect of MNDs on the proprioceptive system. Only a human study conducted by Hammad et al. (2007) to detect the presence of any degree of sensory involvement in patients with ALS. They found that approximately 32% of those patients had affection in the sensory system, about 27% of those patients had abnormalities in the amplitudes of nerve action potentials of the sural sensory nerve, and 91% of those patients had pathological anomalies in the sensory system. Also, they found that large-caliber myelinated fibers got the most affection (73%), while small-caliber myelinated fibers got the least affection (23%). Furthermore, they found that there was a significant degree of degeneration in both axons and myelin sheaths.

### The Mechanism of the Sensation of Muscle Fatigue

The sensation of muscle fatigue is a complex phenomenon. It occurs on both conscious and unconscious perceptions. The sensation of fatigue occurs in the same way by which the body senses any change in its functions, such as the decrease in the heart rate occurs as a consequence to any increase in cardiac output, feeling of an elevated muscle activity rate occurs as a consequence to any elevation in the power generation, which occurs in response to a rise in its physical activity levels, the breathlessness occurs in response to any increase in the ventilation, and the sensation of warm and gummy occurs with any increase in the temperature or sweating (St Clair Gibson et al., 2003).

The sensation of muscle fatigue starts with a change in a particular component during the physical activity, such as a change in sensation of strain in working muscles and/or joints (St Clair Gibson et al., 2003), accumulation of muscle metabolites (Fitts, 1994; Green, 1997), or depletion of substrates (Balsom et al., 1999; McConell et al., 1999). These peripheral changes are sensed by either mechanoreceptors (Pandolf et al., 1975; Mihevic, 1981) or metaboreceptors (Rotto and Kaufman, 1988; Bongiovanni and Hagbarth, 1990). Then, this sensory data reaches the brain to inform it by the level of exertion or fatigue in working muscles (St Clair Gibson et al., 2003).

Mihevic (1981) has demonstrated that the perception of exertion relies on input data from both “muscle and cardiorespiratory system.” This data includes a feedback data about changes in the muscle strain (the primary source for the sensation of fatigue) and a feedback data from the cardiorespiratory system about the depletion in the number of metabolites or substrates (the secondary source of the sensation of fatigue). This study came in accordance with the study of Stamford and Noble (1974), who found that the proprioceptive feedback, precisely from the Golgi tendon organ, was the primary mechanism of the perception of exertion.

Hutton and Nelson (Nelson and Hutton, 1985; Hutton and Nelson, 1986) also conducted two studies to investigate the activity of mechanoreceptors in the fatigued gastrocnemius muscle during ramp stretch in cats. The first study (Mochizuki et al., 2011) investigated the sensitivity of Golgi tendon organs in fatigued gastrocnemius muscle. They found that with ramp stretch, there was a significant decrease in response latencies of Ib nerve types. This decrease presented regardless of any rise in twitch tension or change in peak and static tension. Also, White and Hall (2018) reached the same results and they added that during muscle fatigue the Golgi tendon organs had a tendency to preserve a fixed level of force which could be the cause of the continuous reduction in muscle force.

The second study (Nelson and Hutton, 1985) investigated the sensitivity of muscle spindles in the fatigued gastrocnemius muscle. They found that during static stretching of the fatigued muscle, there was a decline in response latency to any displacement, a rise in the mean frequency, and an increase in resting discharge. While at rest, the frequency of firing to vibration significantly increased in both Ia and IIa nerve fiber types. Also, they found that the sensitivity of cats to different positions significantly decreased with the occurrence of muscle fatigue.

The association between proprioceptive dysfunction and muscle fatigue has been demonstrated in the literature (Ribeiro et al., 2007; Gear, 2011). Ribeiro et al. (2007) investigated the effect of induced muscle fatigue on the knee position sense in elderlies. They performed 30 successive maximal gravity adjusted concentric contractions to knee flexors and extensors using an isokinetic dynamometer. They found that with muscle fatigue, the absolute angular error significantly increased, and the peak torque of knee muscles significantly declined. Gear (2011) investigated the effect of various levels of muscle fatigue of the hamstring muscle on the position sense of the knee joint. In this study, an isokinetic exercise through an angular range of motion used to produce muscle fatigue. Three levels of muscle fatigues were examined, including 90% (mild fatigue), 70% (moderate fatigue), and 50% (maximum fatigue) of hamstring peak torque. He found that there was a significant decrease in the position sense of the knee joint at 90% and 50% of muscle fatigue.

### The Possible Physiological Effects of Proprioceptive Training on Muscle Fatigue in MNDs

It has been reported in the literature that the proprioceptive system has three functions (Jha et al., 2017). It protects joints from excessive and injurious movements via a reflexive mechanism in response to proprioception afferent feedback. It assists in the stabilization of joints during a static posture. Finally, it promotes a better performance of complex movements in more precise coordinated manners. Several studies have stated that proprioceptive training can achieve significant effects on improving motor control dysfunctions in almost musculoskeletal or neurological disorders. These improvements occurred in balance control (Tibone et al., 2013), pain level (McCaskey et al., 2014), motor learning (Aman et al., 2014), and walking parameters (Yong and Lee, 2017).

However, the high value of proprioceptive training in the field of rehabilitation, its effect on decreasing the occurrence of muscle fatigue in patients with MNDs has not been demonstrated yet. The main effect of proprioceptive training on reducing the occurrence of muscle fatigue includes its ability to normalize the firing rate of motor neurons. It has been demonstrated that the decrease or stoppage of the firing of motor neurons has a significant role in reducing the muscular force and developing muscle fatigue (Wan et al., 2017). One of the major causes of the decrease in muscle spindle activity is the decrease in the motor neuron firing rate, which reduces the firing rate of group Ia muscle afferents. Thus, an increase in the presynaptic inhibition and decreasing the firing rate of the motor neurons occur (Brerro-Saby et al., 2008; Vie et al., 2013). Improving the muscle spindle activity by proprioceptive training could help in renormalizing the firing rate of group Ia muscle afferents, presynaptic inhibition and firing rate of motor neurons (Ribot-Ciscar et al., 2000; Hospod et al., 2007; De Luca and Kline, 2012).

Hospod et al. (2007) examined the effect of proprioceptive training on the muscle spindle activity arising from the common peroneal nerve. They found that Ia afferent responses changed significantly after the performance of proprioceptive training. The change in Ia afferent included an increase in the variability of discharge, a decrease in depth of modulation, and a change in spontaneous activity. Potvin and Fuglevand (2017) developed a phenomenological model of motor unit fatigue as a controllable resource to expect muscle fatigue for several tasks and to demonstrate different contractile responses of motor units. This phenomenological model demonstrated that normalization of the firing rate of motor neurons caused an increase in muscle performance and a decrease in the occurrence of muscle fatigue.

Normalization of the firing rate of motor neurons consequently could help in normalizing the amount of calcium released from calcium channels in the sarcoplasmic reticulum and skeletal muscles. The normal release of calcium helps in decreasing the incidence of muscle fatigue because the depletion of calcium is considered one of the main causes of muscle fatigue (Fryer et al., 1995). Muscle spindles activate intrafusal muscle fibers through the activation of gamma motoneurons, which increases strain on the sensory region. Then, through a reflex action intermediated by muscle spindle afferents, an increase in α-motoneurons activity, stimulation of the extrafusal muscle fibers, and occurrence of muscle contraction occur afterward (Edman et al., 2002).

Kuo and Ehrlich (2015) have demonstrated that the contraction of the extrafusal muscle fibers occurs due to the activation of α-motoneurons which stimulates the release of the acetylcholine at the neuromuscular junction. The released acetylcholine spreads across the synaptic cleft and activates nicotinic acetylcholine receptors over the motor endplate. The activation of nicotinic acetylcholine receptors causes an influx of cations (sodium and calcium) then the depolarization of the muscle cell membrane occurs afterward. This depolarization triggers high numbers of voltage-gated sodium channels over the muscle membrane and causes initiation of the action potential.

**FIGURE 1:**
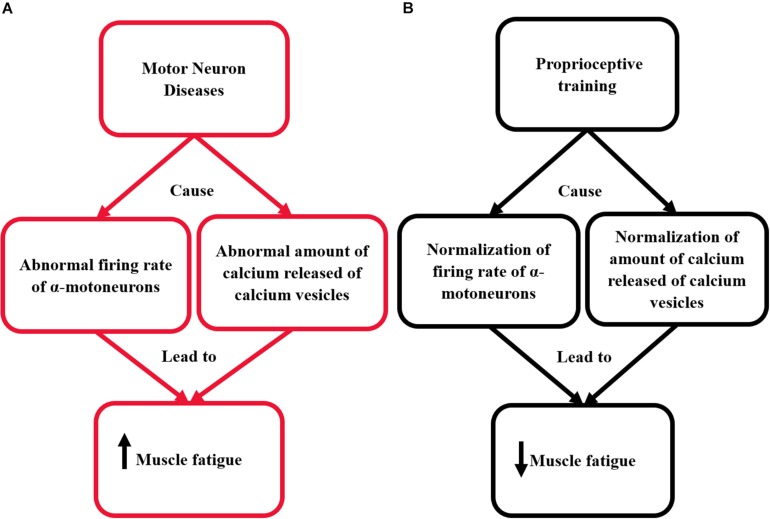
This diagram illustrates the effect of proprioceptive training on re-normalizing the pathological mechanisms responsible for the occurrence of muscle fatigue in patients with MNDs. **(A)** The pathological mechanism responsible for the occurrence of muscle fatigue in patients with MNDs. **(B)** The effect of proprioceptive training on renormalizing the pathological mechanisms responsible for the occurrence of muscle fatigue in patients with MNDs.

The action potential spreads along the surface membrane and transverse tubules. In transverse tubules, the action potential is sensed by the dihydropyridine receptors (voltage-sensor molecules). This mechanism sequentially opens the calcium release channels in the sarcoplasmic reticulum and skeletal muscles. These channels release calcium into the sarcoplasm (Fichna et al., 2015). Then, calcium binds with the troponin to move the tropomyosin far away of the myosin-binding area on actin. This initiates the cross-bridge cycling and muscle contraction (MacIntosh et al., 2012). After muscle contraction, the calcium is removed from the cytoplasm by Ca2+ATPase enzyme. This causes a return of tropomyosin to its blocked location and the relaxation to occur (MacIntosh et al., 2012). Using proprioceptive training could help in the normalization of calcium release mechanism by increasing the muscle spindle activity; this could assist significantly in reducing the incidence of muscle fatigue. The pathological mechanisms responsible for the occurrence of muscle fatigue in patients with MNDs and the effect of proprioceptive training on the renormalization of these mechanisms are illustrated in Figure 1.

### The Effects of Proprioceptive Training to Correct the Imposed Biomechanical Constraints in MNDs

One of the common signs of MNDs is muscle weakness. The pattern of weakness can be either distal to proximal (upper motor neuron disease) or proximal to distal (lower motor neuron disease) (Statland et al., 2015). This weakness occurs due to the degeneration of motor units of certain muscles according to the pattern of weakness for each type. Assuming that force requirements are the same to keep the body erect or produce any movement, thus the load increases in a pattern opposite to the weakness pattern of each type. Thus, movement compensations develop and a further increase in muscle fatigue occurs with any small load or activity.

Several studies (Wu and Ng, 2010; Wu and Shi, 2011; Radovanović et al., 2014; Witiuk et al., 2014; Grunseich and Fischbeck, 2015; Hausdorff et al., 2019) have demonstrated that patients with MNDs have functional movement-compensations. Grunseich and Fischbeck (2015) have demonstrated that patients with SMA usually have progressive leg weakness. This weakness is symmetrical and causes more muscle fatigue in order to keep the balance on uneven surfaces. Radovanović et al. (2014) have demonstrated that patients with ALS have a longer gait cycle and smaller stride length compared to controls. Hausdorff et al. (2019) have shown also that patients with ALS have a longer cycle time and more decrease in cadence, stride length, and velocity compared to controls. They also have shown that patients with ALS spend less time on one leg (swing time) and more time on two legs (double support time).

Functional movement-compensations present in patients with MNDs can be improved by proprioceptive training. Proprioceptive training can adjust the motor control and correct these functional movement-compensations through both increasing patient awareness about the normal movements and correcting the abnormal ones (modulating motor control).

Proprioceptive training can modulate motor control through either central or peripheral mechanisms (Kaya, 2016). Centrally, Eriksson (2001) and Kaya (2016), have shown that proprioceptive training modifies proprioceptive input by modulating muscle spindle control and inducing plastic adjustments in the central nervous system (Vallbo and Al-Falahe, 1990). Peripherally, Hutton and Atwater (1992) have shown that proprioception training causes morphological adaptations in the muscle spindles themselves. These morphological adaptations occur due to micro-adaptations occur to the intrafusal muscle fibers due to some metabolic alterations. Also, these macro-adaptations can occur due to a decline in the response latency of the stretch reflex and a rise in its amplitude.

**FIGURE 2:**
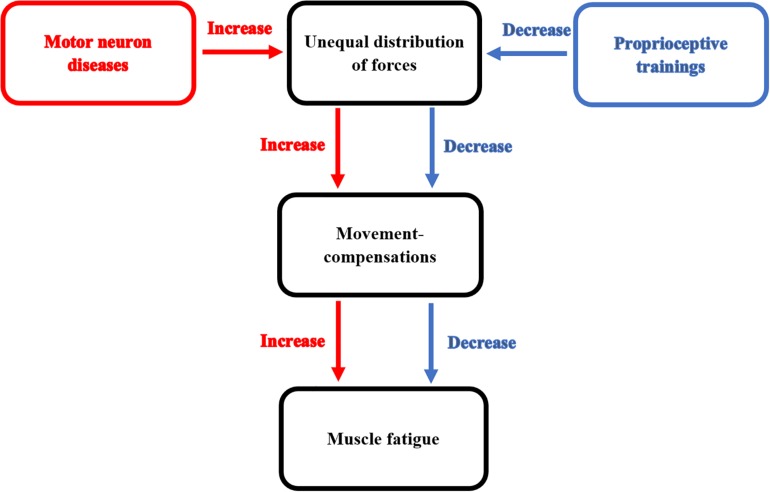
This diagram illustrates the effect of proprioceptive training on decreasing movement-compensations responsible for increasing the occurrence of muscle fatigue in patients with MNDs.

A study conducted by myself and others (Mohamed et al., 2019) to correct the shrug sign which is a type of movement compensations usually develops in patients with adhesive capsulitis. However, adhesive capsulitis is a self-limiting disorder, this sign can prevent its full recovery. In our study, we developed a new proprioceptive training to correct the shrug sign. We found that this proprioceptive training decreased the shrug sign and helped in gaining more shoulder and scapular range of motion. The effects of proprioceptive training on decreasing movement-compensations responsible for increasing the occurrence of muscle fatigue in patients with MNDs are illustrated in Figure 2.

### The Effects of Aging on Pathological Degeneration of Motor and Sensory Neurons Occur in Patients With MNDs

Several studies have shown that the incidence of MNDs among elderlies increases and those patients experience a worse clinical presentation and low survival rate than younger adults (Eisen et al., 1993; Norris et al., 1993; Forbes et al., 2004; Terao et al., 2006; Broussalis et al., 2018). Broussalis et al. (2018) conducted a study to investigate the onset of ALS among elderlies. They found that the majority of admitted patients were elderlies (age of older than 70). Forbes et al. (2004) conducted another study to investigate the clinical presentation of ALS in elderlies over 80 years. They found that clinical presentation and survival rate in elderlies with ALS were worse than younger adults. These results correspond with the study of Terao et al. (2006), who conducted a study to investigate the clinical presentation, and survival rate of ALS among older Japanese patients. They found older patients with ALS had worse survival rates and more complications than younger adults.

Aging is considered one of the key risk factors for the development of MNDs (Kurtzke, 1991; Hospital et al., 1993). Causes of the worse survival rate among older patients with MNDs are not clear yet. These causes might be mainly due to the deteriorating effects of aging on musculoskeletal and neurological systems (Kurtzke, 1991; Hospital et al., 1993). Aging usually causes atrophic changes in extrafusal muscle fibers (McKenzie et al., 2002; Hepple, 2003; Marzetti et al., 2012), degenerations of neuromuscular junctions (Valdez et al., 2010; Tintignac et al., 2015), physiological and cellular modifications in motor axons (Apel et al., 2009; Kang and Lichtman, 2013), and changes in the expression of genes that could critically change normal functions of neuromuscular junctions and skeletal muscles (McKenzie et al., 2002; Weisleder et al., 2006; Jang et al., 2011). Aging causes a decline in both the peripheral and central nervous system processing of sensory information (Nusbaum, 1999). Thus, these mechanisms could significantly cause more complication and worse survival rate among older patients with MNDs.

Furthermore, several studies (Herndon et al., 2002; Pan et al., 2011; Sann et al., 2012; Toth et al., 2012; Li et al., 2016) have examined the effect of age on neuronal tissues using animal models. Understanding these animal models can offer a vision into the bases of selective neuronal susceptibility in neurodegenerative disorders in humans. These animal studies have demonstrated that aging causes abnormal changes in neural axons within the spinal cord. These changes include swelling, waviness, defasciculation, and shrinkage of their diameter (Herndon et al., 2002). Also, aging causes abnormal changes in neurons. These changes include soma distortion, development of abnormal branches, and novel neurite-like projections from the soma (Pan et al., 2011; Tank et al., 2011; Toth et al., 2012). Furthermore, aging causes extensive structural changes in mechanosensory neurons and their microtubule networks (Pan et al., 2011; Toth et al., 2012). These structural changes can disorganize with distorted somas (Pan et al., 2011).

Other types of neurons also exhibit age-related morphological changes, such as dopaminergic neurons, chemosensory neurons, interneurons, and motor neurons. Aging causes morphological changes in the soma of the dopaminergic neuron (McCaskey et al., 2014), axon edging of GABAergic neurons, defasciculation of cholinergic axons in the anterior nerve cord (Pan et al., 2011), and ectopic branches from GABAergic axons (Tank et al., 2011). Aging causes a synaptic decline in the aged neurons, this occurs due to the decrease in the number of synaptic vesicles and size of presynaptic concentrations in the spinal cord (McCaskey et al., 2014).

Aging causes proteins such as SNB-1/synaptobrevin and RAB-3 GTPase, to ectopically collect in synaptic axonal regions and dendrites (Li et al., 2016). Endosomal membrane compartments in aged GABAergic motor neurons disorganize too. These GABAergic motor neurons are important for constructing and reprocessing of synaptic vesicles (Sann et al., 2012). With aging, the presynaptic release of substances decreases in motor neurons and gradually deteriorates afterward, these substances are important for muscle contraction (Liu et al., 2013). Aging causes deterioration of the synaptic organization in the form of a decrease in the number of dendritic spines and the axonal transport, which is vital for synaptic maintenance (Chen and Hillman, 1999; Valdez et al., 2010). With aging, there is a malfunction in neuronal transporters released from synaptic vesicles. The malfunction of these transporters increases the speed of the synaptic decline and motor circuit malfunction. This might explain the chief role of axonal transport in the preservation of synaptic structural integrity through human life (Li et al., 2016).

### The Physiological Effects of Proprioceptive Training to Create Theoretical Bases to Fight the MNDs in Elderlies

However, MNDs significantly affect human life quality and mobility (Simmons, 2013), abnormal structural, and morphological changes occur with aging could aggravate these adverse effects, and speed the degeneration rate occurs to α-motoneurons in patients with MNDs. Normalization of α-motoneurons using proprioceptive training might be a good intervention to fight the occurrence of MNDs in older ages. This can be accomplished by increasing the sensitivity of mechanoreceptors particularly, muscle spindles and Golgi tendon organs which significantly decrease in older ages (Kararizou et al., 2005; Liu et al., 2005; Ribeiro and Oliveira, 2007). It has been demonstrated that increasing the sensitivity of mechanoreceptors could normalize the firing rate of α-motoneurons. This could decrease the disruption of the function of α-motoneurons, the calcium release, and the ATPase enzyme; these mechanisms mainly present with MNDs (Sharma et al., 1995; Ellis et al., 2003; Masson et al., 2014; Nijssen et al., 2017). Thus, using proprioceptive training could be a useful tool to slow down the deterioration in the function of α-motoneurons.

The effectiveness of proprioceptive training on modulating the abnormal tone and improving manual control has been shown with other neuro-degenerative disorders (Shumaker, 1980; Bieñkiewicz et al., 2013; Shih et al., 2016; Wang et al., 2018). Bieñkiewicz et al. (2013) conducted a study to investigate the effect of proprioceptive training by using visual biofeedback on bradykinetic movements of the hand in patients with Parkinson’s disease. They found that temporal features of hand movements significantly moderated by using visual biofeedback. These improvements included an improvement of muscle tone, movement time, and peak velocity.

Shih et al. (2016) examined the effect of using proprioceptive training in the form of game-based training with a Kinect sensor on postural stability in patients with Parkinson’s disease. They found that proprioceptive training-induced improvements in both static and dynamic stability. Wang et al. (2018) studied the effect of using proprioceptive training in the form of game-based training on lower limb function and gait control in patients with spinocerebellar ataxia. They found that proprioceptive training caused an improvement in limb stability, limb-kinetic function, and gait-posture after 4 weeks.

## Conclusion

Proprioceptive training can be an effective method for decreasing the incidence of muscle fatigue in patients with MNDs, particularly elderlies. This can be accomplished through the ability of proprioceptive training to normalize the firing rate of the α-motoneurons and the amount of calcium released from calcium release channels, which has a major role in the occurrence of muscle fatigue. The normalization of α-motoneurons and the amount of calcium released could be helpful to decrease the incidence of development of MNDs or to slow down the progression on presented MNDs in elderlies. Also, proprioceptive training decreases the occurrence of muscle fatigue by correcting the abnormal movement-compensations, which develop due to the biomechanical constraints imposed on patients with MNDs.

## Author Contributions

The author confirms being the sole contributor of this work and has approved it for publication.

## Conflict of Interest

The author declares that the research was conducted in the absence of any commercial or financial relationships that could be construed as a potential conflict of interest.
